# Identification of cuproptosis-related lncRNA for predicting prognosis and immunotherapeutic response in cervical cancer

**DOI:** 10.1038/s41598-023-37898-0

**Published:** 2023-07-03

**Authors:** Xiaoyu Kong, Yuanpeng Xiong, Mei Xue, Jie He, Qinsheng Lu, Miaojuan Chen, Liping Li

**Affiliations:** 1 School of Public Health, Nanchang University, Nanchang, 330006 Jiangxi People’s Republic of China; 2 Department of General Surgery, The First Affiliated Hospital of Nanchang University, Nanchang, 330006 People’s Republic of China; 3 School of Bioscience and Bioengineering, Jiangxi Agricultural University, Nanchang, 330045 Jiangxi People’s Republic of China; 4 Department of Clinical Laboratory, The First Hospital of Nanchang, Nanchang, 330008 Jiangxi People’s Republic of China; 5 Guangzhou Institute of Pediatrics, Guangzhou Women and Children’s Medical Center, Guangzhou Medical University, Guangzhou, 510632 Guangdong People’s Republic of China

**Keywords:** Cervical cancer, Cancer microenvironment

## Abstract

Patients diagnosed with advanced cervical cancer (CC) have poor prognosis after primary treatment, and there is a lack of biomarkers for predicting patients with an increased risk of recurrence of CC. Cuproptosis is reported to play a role in tumorigenesis and progression. However, the clinical impacts of cuproptosis-related lncRNAs (CRLs) in CC remain largely unclear. Our study attempted to identify new potential biomarkers to predict prognosis and response to immunotherapy with the aim of improving this situation. The transcriptome data, MAF files, and clinical information for CC cases were obtained from the cancer genome atlas, and Pearson correlation analysis was utilized to identify CRLs. In total, 304 eligible patients with CC were randomly assigned to training and test groups. LASSO regression and multivariate Cox regression were performed to construct a cervical cancer prognostic signature based on cuproptosis-related lncRNAs. Afterwards, we generated Kaplan–Meier curves, receiver operating characteristic curves and nomograms to verify the ability to predict prognosis of patients with CC. Genes for assessing differential expression among risk subgroups were also evaluated by functional enrichment analysis. Immune cell infiltration and the tumour mutation burden were analysed to explore the underlying mechanisms of the signature. Furthermore, the potential value of the prognostic signature to predict response to immunotherapy and sensitivity to chemotherapy drugs was examined. In our study, a risk signature containing eight cuproptosis-related lncRNAs (AL441992.1, SOX21-AS1, AC011468.3, AC012306.2, FZD4-DT, AP001922.5, RUSC1-AS1, AP001453.2) to predict the survival outcome of CC patients was developed, and the reliability of the risk signature was appraised. Cox regression analyses indicated that the comprehensive risk score is an independent prognostic factor. Moreover, significant differences were found in progression-free survival, immune cell infiltration, therapeutic response to immune checkpoint inhibitors, and IC50 for chemotherapeutic agents between risk subgroups, suggesting that our model can be well employed to assess the clinical efficacy of immunotherapy and chemotherapy. Based on our 8-CRLs risk signature, we were able to independently assess the outcome and response to immunotherapy of CC patients, and this signature might benefit clinical decision-making for individualized treatment.

## Introduction

Cervical cancer is one of the most prevalent reproductive system tumours in females, with rates of occurrence and mortality being particularly high in developing countries^1,2^. Although the development of vaccines against different genotypes of human papillomavirus has helped to reduce the incidence and mortality of cervical cancer, it is still a highly prevalent disease that seriously threatens the health of women^3^. The main treatment options for patients with cervical cancer include surgical removal and radiotherapy, and concurrent chemoradiotherapy is recommended for patients with advanced cervical cancer^4^. Although targeted therapies for cancer treatment have emerged in recent years, with considerable progress, the overall survival rate of cervical cancer is still unsatisfactory^5^. Therefore, there is an urgent need to further investigate the biological mechanisms of cervical cancer progression and to seek new prognostic biomarkers to improve its treatment and prognosis.

Proposed on March 17, 2022, cuproptosis is a unique, copper-dependent modality of regulated cell death with a distinct molecular mechanism and signal transduction pathways^6,7^. Cuproptosis differs from other known modes of cell death, including autophagy, apoptosis, pyroptosis, and ferroptosis. Accumulation of intracellular and mitochondrial copper induces mitochondrial stress, in particular, abnormal aggregation of mitochondrial lipoylated proteins and decreased levels of iron-sulfur cluster proteins, both of which together initiate proteotoxic stress and ultimately lead to cell death^8^. Copper is an indispensable trace element required for maintaining human health that participates in numerous cellular biochemical processes as a cofactor of essential enzymes and has an integral role in mitochondrial respiration and antioxidant and redox metabolism^9^. Nevertheless, high redox activity renders free copper ions highly cytotoxic, and therefore, the intracellular copper availability must be tightly regulated to maintain homeostasis^10^. It has been shown that copper is present at higher levels in the serum of cancer patients than in healthy controls, suggesting that dysregulation of copper homeostasis may affect the development of tumours^11,12^.

Long noncoding RNA (lncRNA) is a form of RNA with a length greater than 200 nucleotides. It has no capability to code for proteins yet affects a variety of biological processes in cells^13^. With the advancement of sequencing technology and widely conducted genome sequencing projects, lncRNAs have become a hot topic for research in recent years^14^. LncRNAs exert a critical role in a variety of biological functions and disease processes, including embryonic development, cell growth and carcinogenesis, by affecting chromatin modification, transcription, and posttranscriptional regulation^15,16^. Due to their tissue-specific expression and high stability, lncRNAs can be detected in body fluids or tumour tissues and are deemed novel potential biomarkers and therapeutic targets for diagnosing diseases, assessing prognosis and tracking disease progression^17^. LncRNAs have also been demonstrated to modulate immunotherapy response by changing the tumour immune microenvironment, particularly tumour immune cell infiltration^18^. For example, lncRNA TCL6 has been linked to tumour-infiltrating lymphocyte infiltration and demonstrated to modulate expression of PD-1, PD-L1 and CTLA-4 immune checkpoint molecules; thus, it can serve as a prognostic and predictive molecular biomarker of breast cancer^19^.

Since serum copper levels are elevated in patients with cervical cancer, altered copper levels are crucial for the development of cancer^20,21^. Hence, modulation of cuproptosis is a prospective therapeutic target for CC. Aberrant expression of lncRNAs is also involved in the progression of CC^22^. As there are few reports on their relationship, we attempted to combine them to investigate the worth of cuproptosis-related lncRNAs in predicting prognosis and response to immunotherapy in CC patients.

For our research, we extracted several cuproptosis-related lncRNAs and then built a signature to predict the outcome of CC patients and proceeded to internal validation and external validation. The results of a series of analyses indicate that this signature can predict prognosis, PFS, level of immune cell infiltration and response to ICI therapy in patients with CC.

## Material and methods

### Data collection

RNA-seq data, mutational data in mutation annotation format (MAF) and corresponding clinical information of cervical cancer samples (306 samples of tumour tissue and 3 samples of adjacent normal tissue) were collected from the cancer genome atlas up to March 30, 2022 (TCGA; https://portal.gdc.cancer.gov/). Patients with missing expression data were omitted from the analysis, with 304 CC patients in the final cohort. According to previously published literature, 19 cuproptosis-related genes were identified (Supplementary Table S1). As an external validation cohort, clinical information and gene expression data for 300 CC patients were collected from the GEO database (GSE44001 https://www.ncbi.nlm.nih.gov/geo/). The immunophenoscore (IPS) for CTLA-4 and PD-1 inhibitors in CC patients was obtained from the cancer immunome atlas up to March 30, 2022 (TCIA; https://tcia.at/home).

### Identification of CRLs in CESE

LncRNA and cuproptosis-related gene expression data were filtered from TCGA-CESE RNA-seq data. The correlation between all lncRNAs and cuproptosis-related genes was calculated via Pearson correlation analysis to determine cuproptosis-related lncRNAs. A lncRNA satisfying correlation coefficient |R^2^|> 0.3 and *P* < 0.001 was defined as a cuproptosis-related lncRNA. The 304 CC samples selected for subsequent analysis were assigned at random into a training group and a test group at a 1:1 ratio utilizing the “caret” package of R software (version 4.1.2).

### Construction and validation of a CRLs signature

By applying univariate Cox regression analysis at *P* < 0.05, CRLs were shown to correlate substantially with the prognosis of CC patients based on survival information encompassing overall survival (OS) and progression-free survival (PFS) in the TCGA database. To avoid overfitting, we employed LASSO and multivariate Cox regression analysis to calculate regression coefficients of significant prognostic lncRNAs in the training group, resulting in the establishment of a prediction signature. Weighting the standardized expression levels of each risk lncRNA with their corresponding regression coefficients generated a risk score (RS) for each patient. The following formula was used: ${\sum }_{i=1}^{n}Coef(i)\times x(i)$, where $Coef(i)$ and $x(i)$ signify the coefficient and standardized expression of lncRNA, respectively. Based on the median risk score of the training group, patients with CC were split into low- and high-risk groups. Kaplan–Meier curves were plotted using the “survival” R package to assess the OS and PFS of the two risk subgroups. To assess survival prediction, a time-dependent ROC curve was used, and the area under the curve (AUC) was computed to test the accuracy and specificity of the CRLs prediction. The GEO dataset was employed as an external validation. We developed a nomogram using the RS and other clinicopathological characteristics and then utilized calibration curves to determine whether the predicted survival probability matches the actual observation. In addition, the clustering ability of the signature was determined by executing the “scatterplot3d” package for principal component analysis (PCA). We further performed Cox proportional hazard regression to evaluate whether the risk signature is able to independently predict prognosis without taking into account other clinicopathological factors. The robustness of the signature was validated in the test group.

### Functional enrichment analysis

With the premise of a false discovery rate (FDR) of 0.05 and |log2FC|> 1, the “limma” R package was applied to determine genes that differ in expression between the high- and low-risk subgroups. To better understand their biological functions and mechanisms, Gene ontology (GO) and Kyoto encyclopedia of genes and genomes (KEGG, www.kegg.jp/kegg/kegg1.html)^23,24^ enrichment analyses of differentially expressed genes (DEGs) were carried out utilizing the “clusterProfiler” and “enrichplot” R packages.

### Tumour microenvironment (TME) and immune cell infiltration analysis

The immunological and stromal components of each CC sample's tumour microenvironment (TME) were analysed by the “estimate” R package to validate variations in microenvironment features across different risk subgroups. Using the “CIBERSORT” R package, the relative percentages of 22 different kinds of human infiltrating immune cells were extracted as well as quantified for each CC sample; samples with *P* values greater than 0.05 were excluded to enhance the accuracy of the estimation results and reveal the relationship between the CRLs signature and infiltration of immune cells.

### Exploring implications of the CRLs signature in clinical treatment

To determine whether the prognostic signature can help predict patient response to immunotherapy, the immunophenoscore (IPS) of CTLA-4 and PD-1 inhibitors in CC patients was obtained from the TCIA database. When differences in IPS between risk subgroups are compared, a higher score indicates a better response rate to ICI immunotherapy. The next step is to observe whether the prognostic signature can be used to predict which drugs will be effective prior to a patient being treated. We applied the “pRophetic” R package to calculate the half maximal inhibitory concentration (IC50) values of chemotherapeutic drugs commonly used in the clinic. The Wilcoxon signed-rank test was used to compare the IC50 values of the high- and low-risk cohorts. A statistically significant *P* value was less than 0.05.

### Quantitative RT–PCR

The human cervical immortalized squamous cell line Ect1/E6E7 and the cervical cancer cell line HeLa were recently acquired from ATCC and grown in DMEM containing 10% foetal calf serum (FCS, Lonza, Ambroise, France). Total RNA was extracted and purified by applying RNAsimple Total RNA Kit (Tiangen Biotech). For reverse transcription, the FastKing RT kit (with gDNase) (Tiangen Biotech) was used. qRT–PCR was carried out using SuperReal PreMix Plus (SYBR Green) from Tiangen Biotech according to the manufacturer's instructions. Each assay was duplicated at least three times for each sample. The 2^(−∆∆Ct)^ method was used to calculate relative expression levels, which were normalized to GAPDH. Gene expression differences were tested by Student’s t test for statistical significance. GraphPad Prism (version 8.0.2) was applied to analyse the results and create graphs (*P < 0.05, **P < 0.01, and ***P < 0.001). The sequences of the primers used are shown in Supplementary Table S2.

### Ethical approval

The study is an analysis of publicly available data and thus did not require ethical approval.

## Results

### Identification of CRLs in CESE

**Figure 1:**
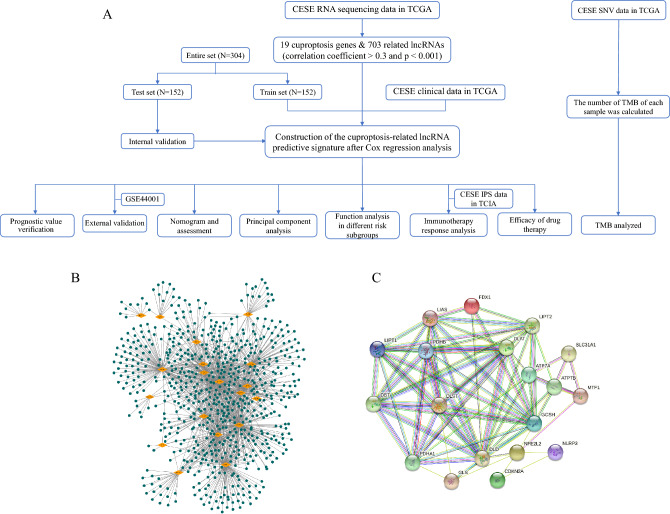
Identification of CRLs. (**A**) Flowchart of the study. (**B**) The co-expression network between cuproptosis-related genes and lncRNAs. (**C**) The PPI network of cuproptosis-related genes.

The overall flowchart of this study is shown in Fig. 1A. A total of 703 lncRNAs were ultimately chosen as CRLs through Pearson correlation analysis between the expression levels of each lncRNA and cuproptosis-related genes (coefficient > 0.3, *P* < 0.001). Then, we created a coexpression network of the cuproptosis-related genes-lncRNAs utilizing Cytoscape_3.9.1 to predict the potential impact of CRLs (Fig. 1B). To show associations between cuproptosis-related genes, a PPI network was mapped with the String website (https://cn.string-db.org/) (Fig. 1C).

### Construction of the CRLs prognostic signature

**Figure 2:**
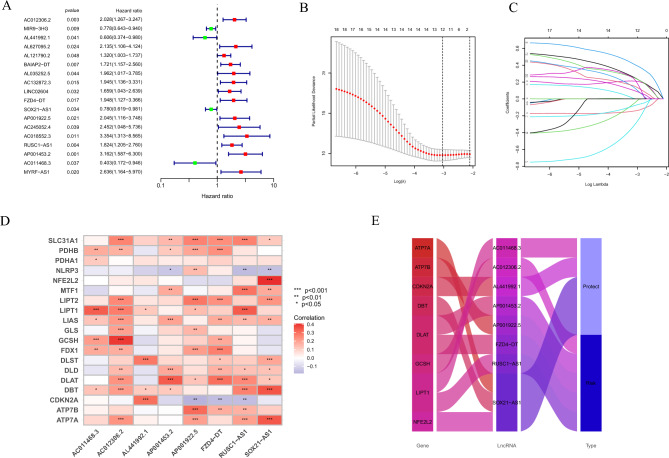
Construction of prognostic signature. (**A**) The forest plot of univariate regression analysis. (**B**) Partial likelihood deviance for the lasso regression. (**C**) LASSO coefficient profiles. (**D**) Heat map. (**E**) Sankey diagram of prognostic CRLs. *P < 0.05, **P < 0.01 and ***P < 0.001.

The 304 CC patients were randomly assigned into two cohorts: the training group (n = 152) and the test group (n = 152). Among clinicopathologic variables, age, grade, and TNM categorization did not differ significantly between the two groups. (Table 1). We performed univariate Cox regression analysis on 703 CRLs based on the training cohort. Eighteen lncRNAs with prognostic significance were initially screened for inclusion in the subsequent analysis (Fig. 2A). To develop a reliable signature for predicting prognostic status, LASSO and multivariate Cox regression analyses were used to filter the lncRNAs (Fig. 2B–C). Eight candidate prognostic CRLs associated with CC prognosis were finally obtained, including three protective lncRNAs (AL441992.1, SOX21-AS1, AC011468.3) and five risk lncRNAs (AC012306.2, FZD4-DT, AP001922.5, RUSC1-AS1, AP001453.2). The heatmap shown in Fig. 2D depicts the relationship between these 8 lncRNAs involved in signature construction and cuproptosis-related genes. The Sankey diagram in Fig. 2E further illustrates the relationship between these prognostic CRLs and the OS of CC patients.

**Table 1:** Clinicopathological features of CC patients in test and train group.

Covariates	Type	Total (n = 304)	Test (n = 152)	Train (n = 152)	*P* value
Age (years), n (%)	≤ 50	186 (61.18)	98 (64.47)	88 (57.89)	0.2895
	> 50	118 (38.82)	54 (35.53)	64 (42.11)	
Grade, n (%)	G1-2	153 (50.33)	76 (50)	77 (50.66)	0.691
	G3-4	119 (39.14)	57 (37.5)	62 (40.79)	
	Unknown	32 (10.53)	19 (12.5)	13 (8.55)	
T, n (%)	T1-2	211 (69.41)	110 (72.37)	101 (66.45)	0.9973
	T3-4	30 (9.87)	15 (9.87)	15 (9.87)	
	Unknown	63 (20.72)	27 (17.76)	36 (23.68)	
M, n (%)	M0	116 (38.16)	61 (40.13)	55 (36.18)	0.664
	M1	10 (3.29)	4 (2.63)	6 (3.95)	
	Unknown	178 (58.55)	87 (57.24)	91 (59.87)	
N, n (%)	N0	133 (43.75)	65 (42.76)	68 (44.74)	0.527
	N1	60 (19.74)	33 (21.71)	27 (17.76)	
	Unknown	111 (36.51)	54 (35.53)	57 (37.5)

**Figure 3:**
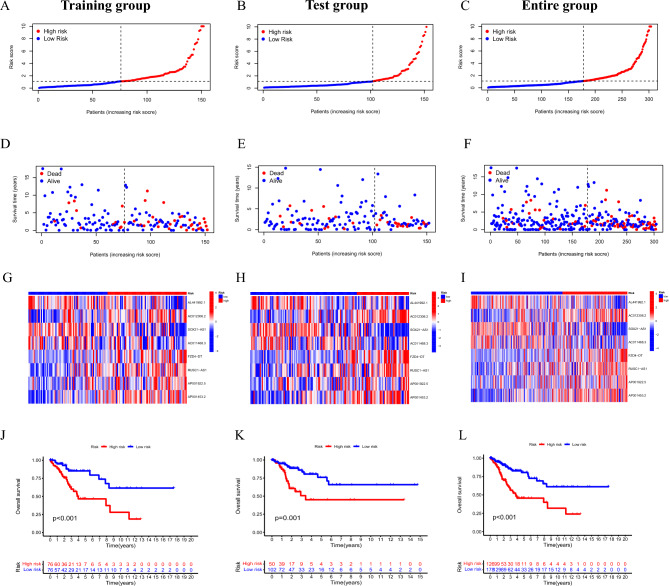
Predictive value of prognostic model in the training, test, entire group. (**A–C**) Risk curve for patients with different RS in the training, test, entire group. (**D–F**) Scatterplots of patients with different survival status and survival time in the training, test, entire group. (**G–I**) Heatmap of the differences in expression of the 8 CRLs between the risk subgroups in the training, test, entire group. (**J–L**) Kaplan Meier survival curves of OS in different risk groups in the training, test, entire group.

To validate the predictive power of a prognostic model consisting of these 8 CRLs to predict CC survival, the following formula was used to determine the RS for each sample: RS = (− 0.495 × AL441992.1 expression) + (− 0.355 × SOX21-AS1 expression) + (− 0.853 × AC011468.3 expression) + (0.556 × AC012306.2 expression) + (0.511 × FZD4-DT expression) + (0.592 × AP001922.5 expression) + (0.468 × RUSC1-AS1 expression) + (0.708 × AP001453.2 expression). The CC patients were classified into different risk subgroups by the median RS. Consistent findings were observed among all subgroups (Fig. 3A–F), with significantly more deaths as the RS increased. The expression level of the 8 CRLs in different risk subgroups is displayed in a heatmap (Fig. 3G–I). Five of them were risk lncRNAs (AC012306.2, FZD4-DT, AP001922.5, RUSC1-AS1, AP001453.2), which were strongly upregulated in the high-risk subgroup. Three were protective lncRNAs (AL441992.1, SOX21-AS1, AC011468.3), which were significantly upregulated in the low-risk subgroup. A significant difference in the prognosis of the two risk subgroups was revealed by a Kaplan–Meier survival plot (Fig. 3J–L, *P* < 0.05). Patients in the low-risk subgroup had markedly greater OS.

**Figure 4:**
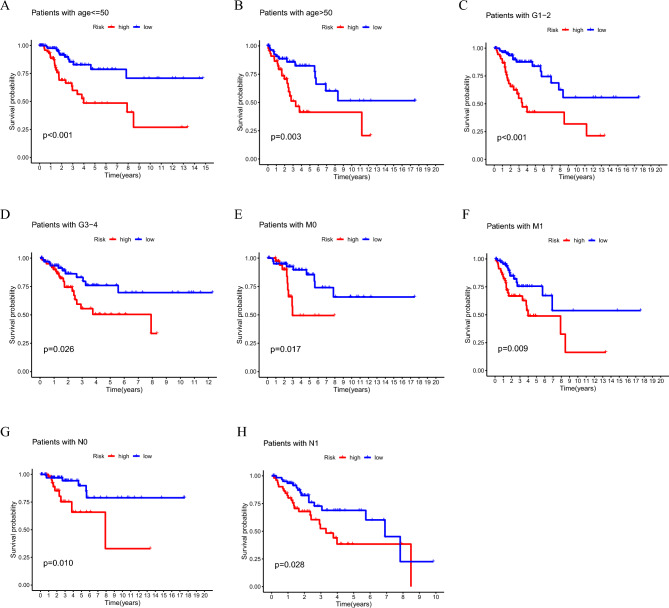
Kaplan Meier survival curves of risk subgroups in patients stratified by different clinicopathological factors. (**A–B**) Age. (**C–D**) Grade. (**E–F**) M stage. (**G–H**) N stage M, metastasis; N, lymph node.

Then, we performed stratified analysis of clinicopathological factors for CC patient prognosis. According to Fig. 4, patients in the high-risk subgroup had considerably shorter OS in clinical stratification analysis based on age, tumour grade, M stage, and N stage. These results demonstrated that the model we established is applicable to different clinical subgroups of patients.

### Predictive performance of the signature evaluated by ROC

**Figure 5:**
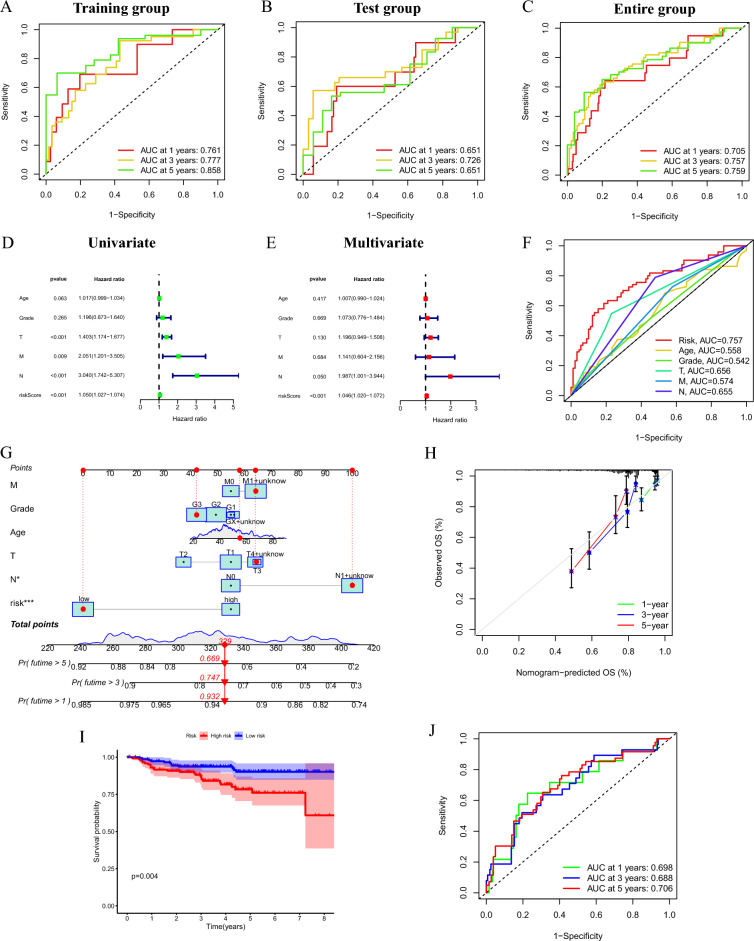
Evaluation of predictive performance, independent prognostic analysis and external verification. (**A–C**) ROC curves of training group, test group and entire group. (**D**) Forest plot of the results of the univariate Cox regression analysis. (**E**) Forest plot of the results of multivariate Cox regression analysis. (**F**) ROC curve of clinicopathological features and risk score, respectively. (**G**) Nomogram. (**H**) Calibration curves. (**I**) The Kaplan–Meier curves of OS in different riskgroups based on GSE44001. (**J**) ROC curves and AUCs at 1-, 3-, and 5-years survival based on GSE44001.

By plotting ROC curves and calculating AUC values, the accuracy of the signature in forecasting the outcome of CC patients was evaluated. AUC values for survival at 1, 3, and 5 years were 0.761, 0.777, and 0.858 for the training group, 0.651, 0.726, and 0.651 for the test group, and 0.705, 0.757, and 0.759 for the entire group, respectively (Fig. 5A–C). These findings demonstrate the good accuracy of the predictive signature we established.

### Independent prognostic analysis and external validation of the 8-CRLs signature

To further assess whether the prognostic signature is an independent prognostic factor for CC patients, we conducted Cox regression analyses of the 8-CRLs signature and conventional clinicopathological factors. The results revealed that tumour T stage, tumour N stage, and risk score correlated highly with OS in univariate Cox regression analysis (*P* < 0.001) (Fig. 5D). Next, multivariate Cox regression analysis revealed that the risk score was an independent factor influencing the prognosis of CC patients (Fig. 5E). The AUC value of the risk score based on the prognostic signature was 0.757, which was higher than that of other clinicopathological features, according to the ROC curve (Fig. 5F). This shows that our model is markedly more effective than other clinicopathological features in predicting patient prognosis. These results confirm the independent predictive value of the 8-CRLs signature.

We next constructed a nomogram incorporating clinicopathological factors as well as the risk score to achieve clinical applicability (Fig. 5G). The calibration curves indicated an excellent match between the observed OS and predicted OS based on the nomogram (Fig. 5H). These results corroborate the accuracy and generalization capability of our developed predictive signature. We were not able to retrieve other datasets that also contained gene expression data for 8 CRLs, clinicopathological features, and survival information for CC patients. Therefore, an external validation cohort was selected from the GEO dataset (GSE44001), which includes survival information of 300 CC patients and expression levels of mRNAs with coexpression relationships with the eight CRLs (Fig. 2E). The KM survival curve indicated that the OS of patients in the high-risk group was notably shorter (Fig. 5I). In ROC analysis, the AUCs were 0.698, 0.688, and 0.706 for 1-, 3-, and 5-year survival rates, respectively (Fig. 5J). Based on all the findings, the 8-CRLs signature has excellent predictive power for predicting the outcome of CC patients.

### The prognostic value of the 8-CLR signature for PFS

**Figure 6:**
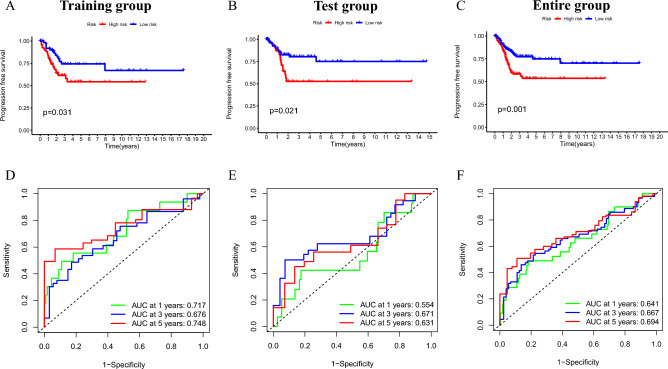
Performance evaluation of model forecast PFS. (**A–C**) Kaplan–Meier progression-free survival curves of training group, test group and entire group. (**D–F**) ROC curves of training group, test group and entire group.

For patients with advanced cancer, a prolonged PFS may signal a reduction in disease burden and symptom alleviation^25^. Taking into account the role of PFS in the clinical outcome of CC patients, our study considered PFS as well, in contrast to most prognostic models that focus primarily on OS. The Kaplan–Meier progression-free survival curves showed that low-risk patients in all three cohorts had more promising PFS (Fig. 6A–C). PFS-based ROC curves were further displayed to confirm the predictive performance of the signature. The AUC values for survival at 1, 3, and 5 years were 0.717, 0.676, and 0.748 for the training cohort, 0.554, 0.671, and 0.631 for the test cohort, and 0.641, 0.667, and 0.694 for the entire cohort, respectively (Fig. 6D–F). These results show that our established signature can accurately predict the PSF of CC patients.

### Principal component analysis (PCA)

**Figure 7:**
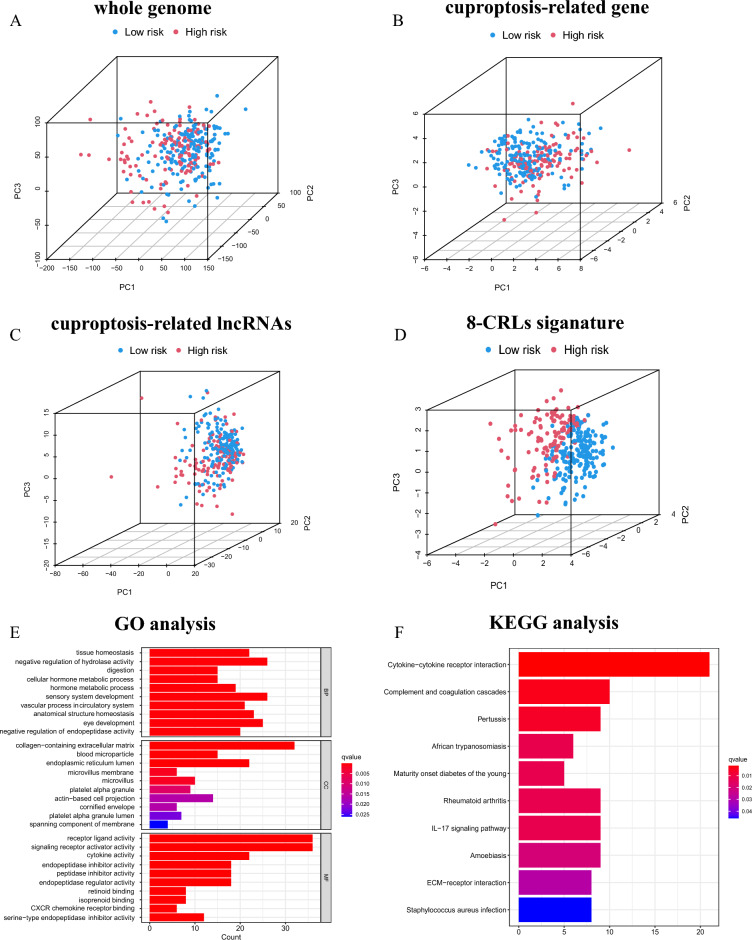
PCA analysis and enrichment analysis. (**A–D**) Distribution of patients based on whole genome, cuproptosis-related gene sets, cuproptosis-related lncRNAs, predictive signature. (**E**) Analysis of DEGs for GO. (**F**) Analysis of DEGs for KEGG (www.kegg.jp/kegg/kegg1.html). Patients in red zone are at high risk, while those in blue zone are at low risk. *PC1* first principal component, *PC2* second principal component, *PC3* third principal component.

To further analyse the clustering capability of the 8-CRLs signature for the distinct distribution pattern of patients in risk subgroups, PCA was performed to visualize the distribution of CC patients between high- and low-risk groups utilizing the whole genome, cuproptosis-related gene sets, cuproptosis-related lncRNAs and eight lncRNAs from the prediction model. The results showed that the lncRNAs included in the prediction model well separated the distribution of patients in different risk subgroups and clearly classified patients into two distinct quadrants (Fig. 7A–D).

### Functional enrichment analysis

We conducted GO and KEGG enrichment analyses of differentially expressed genes (DEGs) based on different risk subgroups to investigate their possible involvement in biological functions and their mechanisms. GO analysis revealed that the DEGs are involved in biological processes (BP) associated with tissue homeostasis and metabolic processes. For cellular components (CC), the DEGs were associated with the extracellular matrix. In the molecular function (MF) category, the DEGs were significantly enriched in receptor ligand activity, signaling receptor activator activity and cytokine activity (Fig. 7E). According to KEGG analysis, the enriched pathways correlate with immune and inflammatory mechanisms, including cytokine–cytokine receptor interaction, complement and coagulation cascades, IL-17 signalling pathway, and ECM-receptor interaction (Fig. 7F).

### TME and immune cell infiltration features in risk subgroups

**Figure 8:**
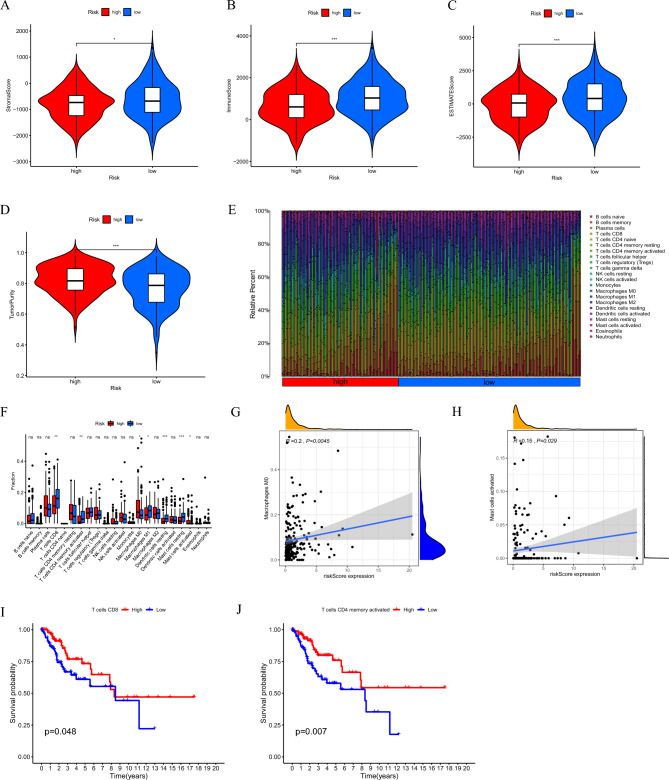
TME and immune cell infiltration features in the risk subgroups. (**A–D**) Comparison of the stromal score, immune score, ESTIMATE score, and tumor purity in two risk subgroups, respectively. (**E**) The composition of 22 types of tumor-immune infiltration cells. (**F**) Immune cells fractions between high and low risk groups in boxplots. (**G–H**) Correlations between risk scores and immune infiltration cells. (**I–J**) Survival analysis of T cell CD8 and T cells memory activated in CC. *P < 0.05, **P < 0.01 and ***P < 0.001.

The TME is composed of various types of cells, including tumour cells, stromal cells, and infiltrating immune cells. Tumour-infiltrating immune cells in the immunological microenvironment are thought to be closely associated with tumorigenesis and cancer progression, and they are a critical factor influencing the therapeutic and prognostic significance of antitumour therapies^26,27^. By applying the ESTIMATE algorithm, we successfully calculated the scores of immune cells, stromal composition, and tumour purity. Our results showed that samples with a low RS had a higher stromal, immune, and ESTIMATE scores as well as lower tumour purity than samples with a high RS, suggesting that the degree of immune infiltration differs between risk subgroups (Fig. 8A–D).

We analysed the relative amounts of 22 different types of immune infiltrating cells. in two risk subgroups using CIBERSORT to further analyse differences in the TME between the risk subgroups. The composition of immune infiltrating cells in samples of the risk subgroup is shown in Fig. 8E. We found some differences between the two subgroups, and we discovered that M0 macrophages and activated mast cells were more abundant in the high-risk subgroup. On the other hand, the low-risk subgroup showed considerably higher levels of CD8^+^ T cells, activated CD4^+^ memory T cells, M1 macrophages, resting dendritic cells and resting mast cells (Fig. 8F). Among them, activated mast cells and M0 macrophages were shown to correlate positively with the risk score, with the remainder correlating negatively (Fig. 8G–H). It is also worth noting that low infiltration of CD8^+^ T cells and activated CD4^+^ memory T cells was significantly linked to poor prognosis (Fig. 8I–J). These findings suggest that abnormal immune cell infiltration may play a role in the carcinogenesis and development of CC and demonstrate that our model has a strong association with the TME and prognosis of CC patients.

### Potential of the 8-CLR signature in immunotherapy and chemotherapy

Immunotherapies have shown promising outstanding therapeutic effects in multiple types of malignant solid tumours^28^. The tumour mutational burden (TMB) and immune checkpoints are two relevant biomarkers for predicting response to immunotherapy and have shown tremendous potential for antitumour effects.

**Figure 9:**
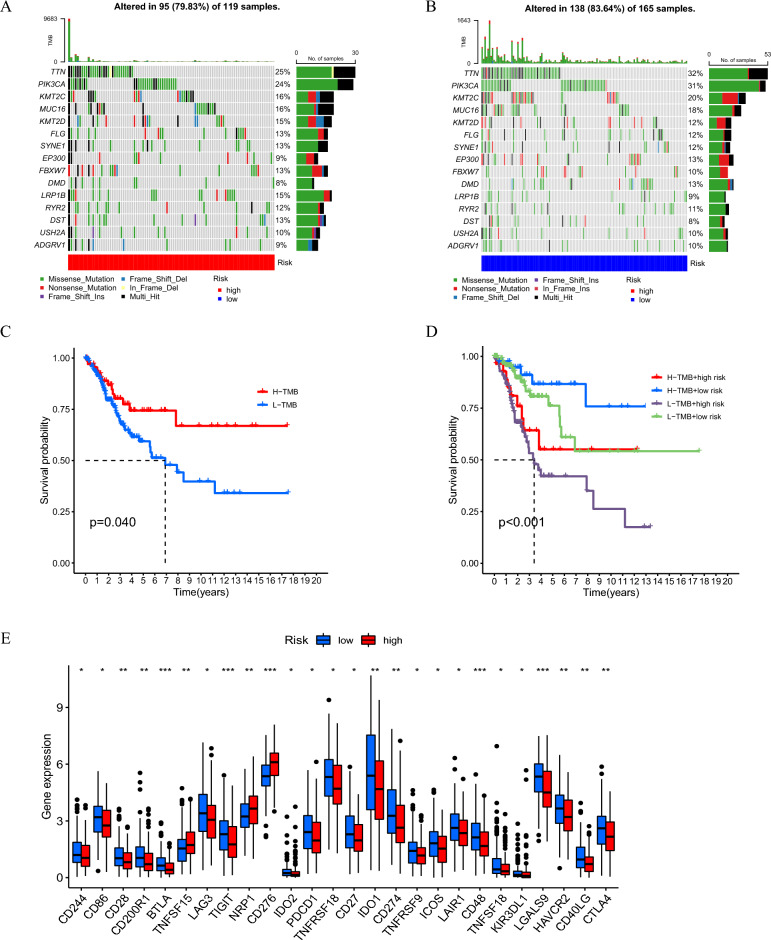
Analysis of TMB and immune checkpoints molecules. (**A–B**) The overall mutation burden of patients in the high- and low-risk groups. (**C**) Prognosis analysis between the low TMB and high TMB patients. (**D**) Stratified survival analyses of TMB-H, high tumor mutation burden; TMB-L, low tumor mutation burden. (**E**) Expression of common immune checkpoints molecules in different risk groups. *P < 0.05, **P < 0.01 and ***P < 0.001.

The TMB is the total number of somatic mutations in each coding region of the tumour cell genome. The TMB between the different risk subgroups is shown in Fig. 9A–B. To clarify the intrinsic association between TMB and patient overall survival, the median TMB was employed as the cut-off point to separate CC patients into low- and high-mutational burden subgroups. Subsequently, we executed survival analysis between the TMB subgroups and discovered that patients with a high TMB had significantly greater survival outcomes than those with a low TMB (Fig. 9C). The results indicate that patients with a high TMB may be more sensitive to immunotherapy than patients with a low TMB. We further performed stratified survival analysis, and the results showed the most favourable prognostic performance for the high TMB/low risk group (Fig. 9D).

Immune checkpoint inhibitors (ICIs), important agents in tumour immunotherapy with prominent clinical benefits, are transforming cancer treatment in every aspect^29^. Overexpression of immune checkpoint molecules suppresses the function of immune cells, thus preventing the body from mobilizing an effective antitumour immune response^30^. Due to the role of immune checkpoints in immunotherapy, we further analysed common immune checkpoint genes between the risk subgroups. Most immune checkpoints such as PDCD 1 appeared to be more activated in the low-risk group (Fig. 9E).

**Figure 10:**
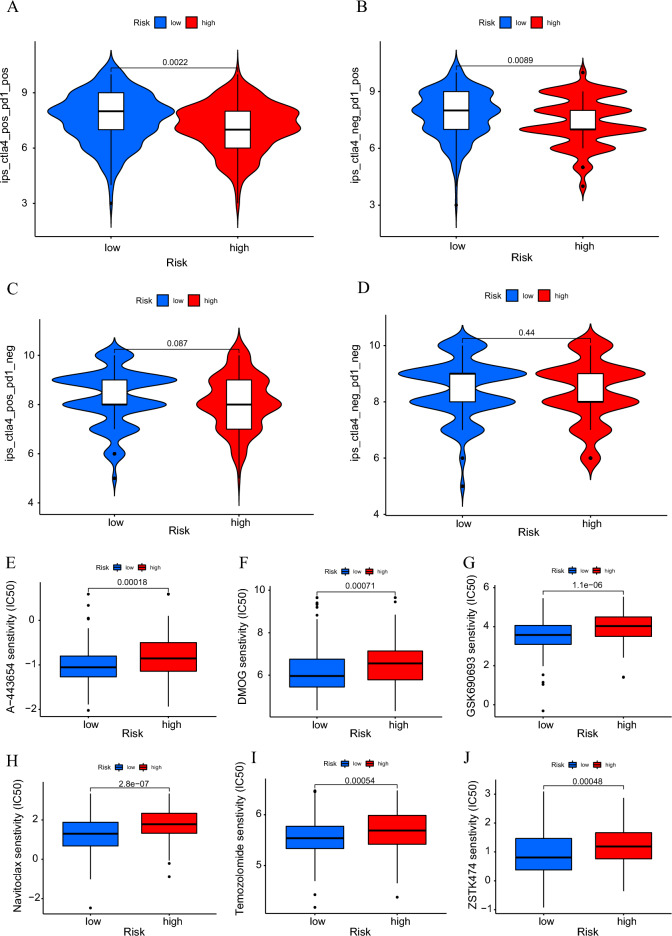
Response to immunotherapy and chemotherapy. (**A–D**) Response to treatment with CTLA-4 and PD-1 inhibitors. (**E–J**) Sensitivity of chemotherapeutic agents A-443654, DMOG, GSK690693, Navitoclax, Temozolomide, and ZSTK474 in the high- and low-risk groups.

The immunophenoscore (IPS) of CTLA-4 and PD-1 inhibitors in 307 CC patients was retrieved from the TCIA database; samples with missing data were eliminated, with 304 samples for subsequent analysis. The results showed that the low-risk group responded better to immunotherapy when treated with either a PD-1 inhibitor alone or a CTLA-4 inhibitor in combination with a PD-1 inhibitor, while there was no statistically significant difference in response to immunotherapy without PD-1 inhibitors between the two groups (Fig. 10A–D). All of these discoveries indicate that patients with a high RS may be less sensitive to immunotherapy with ICIs, according to our prediction model.

Finally, we used the “pRRophetic” R package to assess the value of our prediction model for guiding the clinical use of chemotherapeutic agents in patients with CC by comparing IC50 data for some commonly used chemotherapeutic agents between risk subgroups. Several anticancer drugs showed significantly different IC50 values between the two risk subgroups. Moreover, based on the 8-CRLs signature, the low-risk CC patients were more susceptible to A-443654, DMOG, GSK690693, navitoclax, temozolomide, and ZSTK474, which may be more appropriate for patients with a lower RS (Fig. 10E–J). Our findings suggest that the established model has potential predictive value for chemosensitivity.

### Quantitative real-time reverse transcriptase–polymerase chain reaction analysis

**Figure 11:**
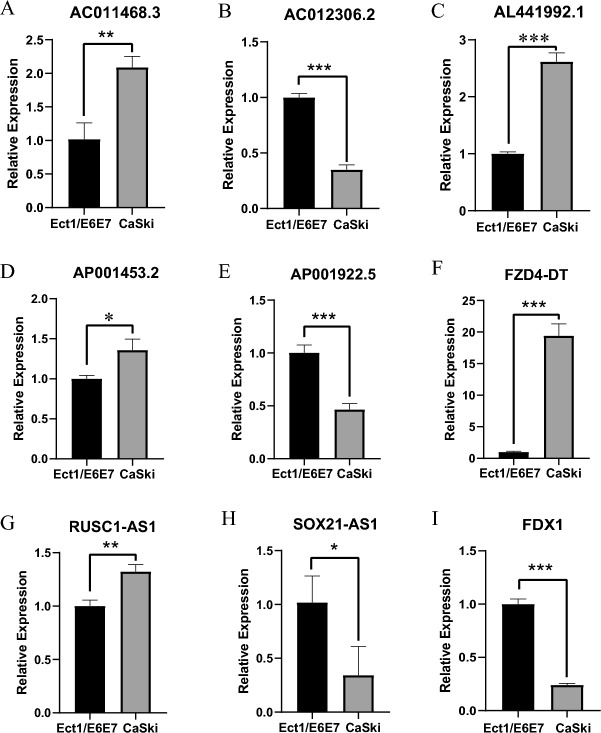
Quantitative polymerase chain reaction detection of 8-CRLs and FDX1 expression in cervical normal cell line and CC cell line. (**A**) AC011468.3. (**B**) AC012306.2. (**C**) AL441992.1. (**D**) AP001453.2. (**E**) AP001922.5. (**F**) FZD4-DT. (**G**) RUSC1-AS1. (**H**) SOX21-AS1. (**I**) FDX1. *P < 0.05, **P < 0.01 and ***P < 0.001.

Levels of the 8 cuproptosis-related lncRNAs (AC011468.3, AC012306.2, AL441992.1, AP001453.2, AP001922.5, FZD4-DT, RUSC1-AS1, SOX21-AS1) were assessed in Ect1/E6E7 and HeLa cells. As depicted in Fig. 11A–H, the expression levels of these lncRNAs differed significantly between the two cell lines. This indicates that these lncRNAs are significantly differentially expressed between tumour and normal cervical cells. Moreover, we examined expression levels of key genes for the cuproptosis process in the two cell lines (Fig. 11I). And the results of qRT-PCR are for reference purposes only. Overall, the results of our experiments support the robustness of our model.

## Discussion

Regulatory cell death (RCD) was discovered as a type of cell death mediated by activation of one or more signalling pathways and is critical for normal cell growth and the immune response, and evasion of RCD is one of the key hallmarks of cancers^7,31^. Cuproptosis is a copper-dependent form of RCD characterized by aberrant aggregation of mitochondrial lipoylated proteins and decreased levels of iron-sulfur cluster proteins caused by copper accumulation. Under normal conditions, intracellular copper ion concentrations are maintained at low basal levels through a set of homeostatic mechanisms^32^. Exogenous copper ions are transported into the cell using copper ionophores, which are small molecular compounds that bind copper ions; this leads to elevation of intracellular copper levels, particularly in the mitochondria, resulting in excessive cell respiration and cytotoxicity and consequently cell death^33^. It has been reported that inducing cuproptosis in cancer cells has tremendous research potential in terms of suppressing tumour progression.

As high-throughput sequencing technology becomes widely available, a growing number of research have been carried out to predict the prognosis of cervical cancer patients by the alteration of a single lncRNA or combined expression of several lncRNAs in body fluids^34^. For instance, downregulated expression of the lncRNA GAS5 predicts worse outcomes in patients with CC^35^. Studies have demonstrated that lncRNAs are engaged in tumour initiation and progression by affecting tumour immune responses and immune cell infiltration; therefore, lncRNAs are potential biomarkers and targets for antitumour therapy^18,36^.

In this research, we selected 703 cuproptosis-associated lncRNAs from TCGA-CESE transcriptomic data by Pearson correlation analysis based on 19 cuproptosis-associated genes. Then, 18 cuproptosis-related lncRNAs associated with CC prognosis were identified by univariate Cox regression, and after LASSO and multivariate Cox regression, a risk prediction model containing AL441992.1, SOX21-AS1, AC011468.3, AC012306.2, FZD4-DT, AP001922.5, RUSC1-AS1, and AP001453.2 was constructed. There have been several previous reports on the establishment of cuproptosis-related lncRNA models for predicting the prognosis of cervical cancer^37–39^ (Table 2). Compared to a study by Liu, Wang et al. using only the TCGA database, we added the GEO database (GSE44001) as an external validation to make the obtained results more persuasive. Our model incorporated new lncRNAs (AC011468.3, AC012306.2, AL441992.1, AP001453.2, AP001922.5, FZD4-DT), and we further explored differential expression of lncRNAs in the model as well as the key cuproptosis gene FDX1 in cell lines by qRT–PCR. When evaluating survival predictions, AUCs at 1, 3 and 5 years were 0.705, 0.757 and 0.759 for the entire group, respectively, which were significantly higher than those of previous studies. Our signature was also valuable for predicting PFS in CC patients. It is also worth mentioning that we used IPS data obtained from the TCIA database to assess response to immunotherapy with ICIs in patients in different risk groups. As demonstrated by the calibration curves, we have a greater consistency between the actual survival and the predicted survival of the nomogram based on our signature, which is more beneficial for clinical applications. In summary, our prognostic model has good and stable prognostic prediction ability.

**Table 2:** Main characteristics of the previous related studies.

Authors	Year	Database	Sample size		CRL signature	AUC		
			Training cohort	Testing cohort		Training cohort	Testing cohort	Entire cohort
Liu et al.	2023	TCGA	152	152	AC063943.1,CDKN2B-AS1, CNNM3–DT	Not available	Not available	0.699, 0.679, 0.698 (1, 3, and 5 years)
Liu et al.	2022	TCGA	143	142	AC009902.2, AL354733.3 , AL441992.1, LINC01305, AL354833.2, CNNM3-DT, SCAT2	0.807, 0.824, 0.793 (1, 3, and 5 years)	0.652, 0.676, 0.669 (1, 3, and 5 years)	0.724, 0.757, 0.741 (1, 3, and 5 years)
Wang et al.	2022	TCGA	152	152	AC096992.2, AJ003147.1, SOX21-AS1, AL049869.2, CNNM3-DT, ARHGAP31-AS1	Not available	Not available	0.718, 0.713, 0.646 (1, 3, and 5 years)

According to previous studies, the lncRNA SOX21 antisense RNA 1 (SOX21-AS1) can alleviate oxidative stress and inhibit neuronal apoptosis in Alzheimer's disease mice and is associated with disease development^40^. Our qRT–PCR results showed its lower expression in a cervical cancer cell line, which may represent an association with good outcome. In patients with lung squamous cell carcinoma, the low AC011468.3 expression group achieved significantly longer recurrence-free survival than the high AC011468.3 expression group^41^, and our qRT–PCR results showed it to be highly expressed in CC cells. Expression of AC012306.2 was found to correlate positively with poor patient prognosis in a cervical cancer investigation, with the 5-year survival rate being significantly lower among patients with high expression of AC012306.2 than among other patients^42^. To further investigate its expression in cervical cancer, we performed qRT–PCR, and the results suggest that it is poorly expressed in CC cell lines. RUSC1-AS1, which is located on chromosome 1q22, has been shown to promote the development of CC through a competitive endogenous RNA (ceRNA) mechanism, sponging microRNA-744 and upregulating Bcl-2 expression^43^. Furthermore, RUSC1-AS1 promotes development of hepatocellular carcinoma by modulating the miR-340-5p/CREB1 axis to affect the proliferation, invasion and migration abilities of cancer cells^44^. In agreement with this, our qRT–PCR results confirm that it is highly expressed in CC cell lines. Moreover, AP001453.2 was found to be closely associated with hypoxia in triple-negative breast cancer tissues and substantially expressed in the group with a high hypoxia fraction based on RNA-seq data from TCGA-TNBC^45^. Our qRT–PCR results showed high expression of AP001453.2 in CC cells. In an immune-related lncRNA-based prognostic prediction model for CC patients, AL441992.1 was discovered to be less expressed in the high-risk subgroup^46^. This was consistent with our qRT–PCR results. In contrast, FZD4-DT and AP001922.5 have rarely been reported in clinical or basic research. Therefore, we verified differential expression of these two genes at the cellular level, and the results are shown in Fig. 11E–F. FZD4-DT was highly expressed in the cervical cancer cell line, consistent with its characterization as a risk gene; however, the risk gene AP001922.5 was expressed at low levels in the cervical cancer cell line, which may need to be further explored. According to the latest related research, FDX1 converts Cu^2+^ to more toxic Cu^1+^, which makes it a key positive regulator of cuproptosis^8^. Therefore, we additionally detected differences in expression at the cellular level via qRT–PCR, and the experimental results showed low expression of FDX1 in a cervical cancer cell line, which was in accordance with our expectation. Finally, it is important to note that the cell line used for qRT-PCR is a single cervical cancer cell line, so the qRT-PCR validation results are for reference purposes only. Based on a Sankey diagram, these lncRNAs are associated with regulatory genes specifically related to cuproptosis metabolic pathways. AL441992.1 is associated with CDKN2A, a negative regulator of cell cycle progression that is considered to be involved in cuproptosis metabolic pathways^47^. DLAT, as a molecule encoding mitochondrial proteins involved in the catabolic glucose pathway, is associated with cellular energetics and energy metabolism reprogramming and may also be linked to FZD4-DT^48^. ATP7B encodes a copper transporter critical for cellular copper homeostasis^49^, and its mutation leads to the development of copper metabolism disorders, possibly regulated by AP001922.5.

To assess the prognostic value of our 8-CRLs signature for predicting OS and PFS in CC patients as well as its effectiveness in assessing response of CC patients to immunotherapy with ICIs, CC patients were randomly allocated to training and test cohorts at a 1:1 ratio. Significantly shorter overall survival times were observed for CC patients in the high-risk group in the training, test and entire cohorts. These prognostic differences between two risk subgroups based on different clinical characteristics were clearly illustrated using Kaplan–Meier survival analysis, including age groups, G1-2/G3-4 stage groups, M0/M1 stage groups, and N0/N1 stage groups. According to ROC curves, the signature showed good prediction effectiveness in the training cohort, test cohort, and entire cohort. The AUC values of the model were greater than 0.7 at 1, 3, and 5 years. The remarkable capacity of our model to distinguish between high- and low-risk groups in all samples was clearly shown by PCA.

Our prognostic signature was also linked to infiltration of immune cells in the TME and immune-related biomarkers to predict the clinical outcome of immunotherapy. We discovered that the risk subgroups have diverse tumour microenvironments, which may lead to differences in prognosis and response to immunotherapy. For samples with a high RS, the stromal score and immune score were lower, and the tumour purity was higher. Levels of M0 macrophages and activated mast cells were higher in the TME of high-RS samples. These aberrantly infiltrated immune cells may be associated with the advancement and poor prognosis of cervical cancer. It is undeniable that CD8^+^ T cells play a key role in the body's antitumour immunity. Consequently, reduced levels of CD8^+^ T cells in the high-risk subgroup may lead to poor responses to immunotherapy^50^.

ICIs immunotherapy consisting of anti-CTLA4 and anti-PD1 has been shown to be effective in non-small cell lung cancers and oropharyngeal cancer, and the feasibility of applying immunotherapy to cervical cancer is receiving increasing attention and research^51,52^. ICIs act on immune checkpoints and are used to enhance antitumour immunity or to increase immunosuppression. According to our prediction model, the majority of immune checkpoints in the low-risk group showed higher activation, suggesting that CC patients in the low-risk group may be more sensitive to immunotherapy than those in the high-risk group (Fig. 9E). As shown in Fig. 10A–D, patients in the low-risk group were more sensitive to treatment with ICIs, suggesting that they are more likely to benefit from immunotherapy. Nevertheless, there is currently no thorough and systematic study on the relationship among cuproptosis, the immune microenvironment and immunotherapy in CC. Therefore, the goal of our research was to discover appropriate immunotherapy targets and prognostic biomarkers.

However, we admit that our research has certain limitations. First, this was a retrospective study using data from the TCGA database. Therefore, some discrepancies may exist. Second, another limitation of this study was the reliance on a single cervical cancer cell line. We will subsequently include more samples and cell lines for validation when available. Third, the mechanism of function of cuproptosis-related lncRNAs requires further experimental demonstration, and the validity of the 8-CRLs signature established in this study needs additional confirmation in clinical trials with large samples.

As mentioned above, the 8-CRLs signature was found to be efficient in independently predicting the prognosis of CC patients. This study will serve as a foundation for future research on the mechanism and clinical therapeutic effectiveness of cuproptosis-related lncRNAs in CC, but further experimental validation is still needed.

## Conclusion

The 8-CRLs signature established in this study is beneficial for predicting the prognosis and therapy response in CC patients and may provide unique insights into cancer treatment.

## Supplementary Information

Supplementary Tables.

## Data Availability

The data used to support findings of this study are available from the corresponding author upon request.

## Abbreviations

CC: Cervical cancer
lncRNA: Long non-coding RNA
CRL: Cuproptosis-related lncRNA
TCGA: The cancer genome atlas
TCIA: The cancer immunome atlas
DEGs: Differentially expressed genes
ROC: Receiver operating characteristic
AUC: Area under curve
FDR: False discovery rate
OS: Overall survival
PFS: Progression-free survival
GO: Gene ontology
KEGG: Kyoto encyclopedia of genes and genomes
PCA: Principal component analysis
TMB: Tumor mutation burden
TME: Tumor microenvironment
IPS: Immunophenoscore
ICIs: Immune checkpoint inhibitors
IC50: Half maximal inhibitory concentration
RCD: Regulatory cell death
