# Supplementary material for: Identification of cuproptosis-related lncRNA for predicting prognosis and immunotherapeutic response in cervical cancer
Source: Sci Rep. 2023 Jul 3;13:10697. doi: 10.1038/s41598-023-37898-0 (PMC10318051; doi:10.1038/s41598-023-37898-0)
Supplement: Supplementary file 1 — Supplementary Tables. [file 41598_2023_37898_MOESM1_ESM.docx]

**SUPPLEMENTAL MATERIALS**

**Identification of cuproptosis-related lncRNA for predicting prognosis and immunotherapeutic response in cervical cancer**

Xiaoyu Kong1†, Yuanpeng Xiong2†, Mei Xue3, Jie He4, Qinsheng Lu5, Miaojuan Chen5*, Liping Li4*

1. School of Public Health, Nanchang University, Nanchang, 330006, Jiangxi, China

2. Department of General Surgery, The First Affiliated Hospital of Nanchang University, Nanchang 330006, China

3. School of Bioscience and Bioengineering, Jiangxi Agricultural University, Nanchang, 330045, Jiangxi, China

4. Department of Clinical Laboratory, the First Hospital of Nanchang, Nanchang, 330008, Jiangxi, China

5. Guangzhou Institute of Pediatrics, Guangzhou Women and Children's Medical Center, Guangzhou Medical University, Guangzhou, 510632, Guangdong, China

†Xiaoyu Kong and Yuanpeng Xiong contributed equally to this work.

*Correspondecnce: Liping Li, Department of Clinical Laboratory, the First Hospital of Nanchang, Jiangxi, Nanchang, 330008, P.R.China

E-mail: liliping-2012@hotmail.com; soso1010@126.com

| **Table S1.** Cuproptosis-related genes |
| --- |
| NFE2L2 |
| NLRP3 |
| ATP7B |
| ATP7A |
| SLC31A1 |
| FDX1 |
| LIAS |
| LIPT1 |
| LIPT2 |
| DLD |
| DLAT |
| PDHA1 |
| PDHB |
| MTF1 |
| GLS |
| CDKN2A |
| DBT |
| GCSH |
| DLST |

**Table S2.** Primer sequences

| Gene id | | Primer F | | Primer R |
| --- | --- | --- | --- | --- |
| AL441992.1 | | 5′-GTGAGTCCGGGAGGCAAAAT-3′ | | 5′-CTGTCACCACAGAGCAACCT-3′ |
| SOX21-AS1 | 5′-GCTGCGGCACATTTAGAACATGATC-3′ | | 5′-CCACAGGGCTAGGTCAAATCCAAAC-3′ | |
| AC011468.3 | 5′-GACGCAGGAGGAACACTTGAACC-3′ | | 5′-GGCAACGGAATAGGCTCTGTCTG-3′ | |
| AC012306.2 | 5′-CCCTGTGTGTTGTTGGTAGAGATGG-3′ | | 5′-TGTGCCTTGTGGTATGTAACTGTGG-3′ | |
| FZD4-DT | 5′-GCCAGTGGCCCTCTAAGTTT-3′ | | 5′-TGTGACACAGCCCATCTGAC-3′ | |
| AP001922.5 | 5′-CCAGCAGTAGCCACCAAGATGTAC-3′ | | 5′-ATGATTGAGCCGAAGCACAGGATG-3′ | |
| RUSC1-AS1 | 5′-CCCTCTCGTCCTCTTCCTCCAAG-3′ | | 5′-GCTTCTGCTGCTCTCCTGATTCC-3′ | |
| AP001453.2 | 5′-GAACTGTGCTCTGTGCCATCTCC-3′ | | 5′-CCACCTGCTTCTCACTCTGTCATTG-3′ | |
| FDX1 | 5′-TTC​AAC​CTG​TCA​CCT​CAT​CTT​TG-3′ | | 5′-TGC​CAG​ATC​GAG​CAT​GTC​ATT-3′ | |
